# From Risk to Resilience: Multidimensional Influences on Health and Well-Being in Later Life

**DOI:** 10.1093/geroni/igaf122.1361

**Published:** 2025-12-31

**Authors:** Ann Nguyen, Katrina Ellis

## Abstract

This symposium examines risk and resilience factors that shape the health and well-being of middle-aged and older adults using a health equity lens. The first paper reports the findings for the association between stressful social roles and poor sleep quality among women experiencing menopause. The findings demonstrate that stress from social roles negatively impacts sleep health. The second paper investigates how the interplay between neighborhood characteristics and education impacts cognitive decline. The results indicate that the combination of low educational attainment and living in a disadvantaged environment is a risk factor for cognitive decline. The third paper examines the financial exploitation among people living with dementia using a scoping review methodology. The results identify risk and protective factors and intervention strategies for financial exploitation. The fourth paper interrogates the relationship between resilience and health in older African Americans living with advanced chronic kidney disease. The findings indicate that resilience may be a response to managing multiple health conditions. The fifth paper examines the interactive effect of social support and depressive symptoms on mortality risk among older adults living with type 2 diabetes. The results demonstrate the importance of leveraging social support and managing depressive symptoms in diabetes management. Overall, these papers will provide important insights into modifiable psychosocial factors that influence health and well-being in later life. Modifiable psychosocial factors have broad translational importance in promoting health equity. The findings will be discussed with attention to important socio-cultural and contextual factors and highlight implications for gerontological practice and research.

